# Curcumin mitigates acrylamide‐induced ovarian antioxidant disruption and apoptosis in female Balb/c mice: A comprehensive study on gene and protein expressions

**DOI:** 10.1002/fsn3.4076

**Published:** 2024-03-07

**Authors:** Sanaz Alaee, Zahra Khodabandeh, Mahintaj Dara, Elham Hosseini, Mona Sharma

**Affiliations:** ^1^ Department of Reproductive Biology, School of Advanced Medical Sciences and Technologies Shiraz University of Medical Sciences Shiraz Iran; ^2^ Stem Cells Technology Research Center Shiraz University of Medical Sciences Shiraz Iran; ^3^ Department of Obstetrics and Gynecology, Mousavi Hospital, School of Medicine Zanjan University of Medical Sciences Zanjan Iran; ^4^ Zanjan Metabolic Diseases Research Center Zanjan University of Medical Sciences Zanjan Iran; ^5^ Department of Reproductive Biology AIIMS New Delhi India

**Keywords:** acrylamide, antioxidants, curcumin, female infertility

## Abstract

Curcumin is known for its antioxidant properties. This study aimed to investigate the impact of curcumin on acrylamide (ACR)‐induced alterations in the first‐line antioxidant defense of ovarian tissue. Female Balb/c mice were divided into control, ACR (50 mg/kg), ACR/CUR100 (received Acr + curcumin100 mg/kg), and ACR/CUR200 (Acr + curcumin 200 mg/kg) groups, and received oral treatments for 35 days. Evaluation of antioxidant enzyme expression (*Sod*, *Cat*, *Gpx* genes), pro‐apoptotic gene expressions (*Bax*, *Caspase 3*), and anti‐apoptotic gene expression (*Bcl2l1*) at mRNA and protein levels was done. Percentage of apoptotic cells using Terminal deoxynucleotidyl transferase dUTP nick end labeling (TUNEL) assay was performed. The model group (ACR) showed decreased mRNA expression of *Sod*, *Cat*, and *Gpx* genes compared with the control group. Treatment with two different doses of curcumin (CUR100 and CUR200) significantly increased *Sod*, *Cat*, and *Gpx* gene expression, with CUR200 demonstrating significant recovery. SOD, CAT, and GPX protein levels were similar to mRNA expression trends, significantly increased with curcumin administration. Acrylamide exposure significantly increased *Bax* and *Caspase 3* expression and decreased *Bcl2l1* gene expression leading to a notable rise in apoptosis in ACR group as compared to the control group. Conversely, curcumin administration, significantly reduced *Bax* and *Caspase 3* expressions, with an increase in *Bcl2l1expression*, though not statistically significant. TUNEL assay revealed a substantial decrease in apoptosis in curcumin‐received groups. In our study, ACR exposure adversely affected ovarian antioxidant defense thereby leading to increased pro‐apoptotic markers. Notably, curcumin treatment effectively mitigated these effects, restored antioxidant potential, and reduced acrylamide‐induced toxicity in female mouse ovaries.

## Graphical abstract

The issue of whether and how acrylamide intake affects human health and fertility status, has recently attracted considerable attention. The purpose of this study was to determine whether curcumin treatment, as a potential antioxidant, impacts ACR‐induced alterations in the first‐line antioxidant defense and apoptosis in the mouse ovarian tissues.

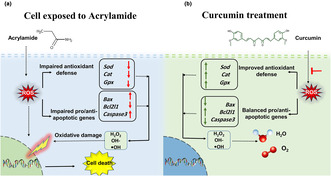

## 1 BACKGROUND

Acrylamide (ACR) is a chemical compound with various applications in industries and frequently used for producing laboratory materials (Duan et al., 2015). Dietary exposure is the main route for acrylamide consumption in humans (Matoso et al., 2019). According to previous studies, ACR is formed in carbohydrate‐rich foods during preparation at a high temperature (about 120°C) through a sequence of non‐enzymatic reactions known as the “Maillard reaction,” that occurs between reducing sugars and free amino acids (Aldawood et al., 2020; Bachir et al., 2023). Because of its low molecular weight and high solubility, ACR may pass through a variety of cellular membranes as reported in humans and animals (Yilmaz et al., 2017). Some previous studies showed that ACR accounts for one of the major health concerns and has been linked to damage to both male and female reproductive organs (ALKarim et al., 2015; Tyl & Friedman, 2003). Animals exposed to acrylamide may have decreased fertility, implantation abnormalities, and lower postnatal survival (ALKarim et al., 2015; Duan et al., 2015; Tyl & Friedman, 2003). Pekmezci and Basaran (2023) have shown that the exposure to acrylamide in pregnant women leads to an increased risk of cancer.

Dietary acrylamide consumption was linked to changes in sex hormones in women (Hogervorst et al., 2013). Acrylamide exerts an impact on the quality of oocytes by influencing cytoskeletal integrity, abnormal mitochondrial distribution, generating reactive oxygen species (ROS), initiating apoptosis, and causing epigenetic modifications (Duan et al., 2015). In recent years, the World Health Organization has recognized acrylamide as a global concern, classifying it as a reproductive toxin (Kashani et al., 2021). In a recent investigation, the cytotoxicity induced by ACR has been consistently associated with oxidative stress (Ibrahim et al., 2020). The cytotoxic impact of ACR may trigger ROS formation, leading to cytotoxic and genotoxic consequences through alterations in the cellular redox state (Gao et al., 2023). Research has shown that the bioactive constituents present in certain plants possess the capacity to alleviate oxidative stress (Alaee et al., 2023; Bolouki et al., 2020; Mbiba Hassanu et al., 2022; Mohammadi et al., 2023; Obinna & Agu, 2023; Ojatula, 2021; Oyetunji et al., 2021; Yaghutian Nezhad et al., 2021). Hence, it appears that antioxidant molecules might aid in mitigating the deleterious effects of this compound.

Curcumin, characterized by the chemical formula 1,7‐bis(4‐hydroxy‐3 methoxyphenyl)‐1,6‐heptadiene‐3,5‐dione, represents the principal bioactive compound found in *Curcuma longa* (El‐Desoky et al., 2022). Evidence has shown that curcumin exhibits anti‐inflammatory, antioxidants, anti‐cancer activities (Khajeh pour et al., 2023; Mailafiya et al., 2023). These advantageous features are attributed to its elevated antioxidant activity, stemming from the hydroxyl and methylene groups within the β‐diketone moiety present in its chemical structure (Chen et al., 2023; Wang et al., 2017). There are several studies that explored the positive effects of curcumin on the ovary tissue. Azami et al. (2020) showed that curcumin could prevent ovarian aging by increasing ovarian volume and the number of follicles. Lv et al. (2021) also suggested that curcumin had beneficial effects on the ovary and reproductive organs by regulating the PTEN‐AKT‐FOXO3a pathway. Furthermore, ACR exposure led to a significant increase in estradiol, luteinizing hormone, testosterone, ovarian tumor markers such as CA125, and carcinoembryonic antigen (CEA), while serum progesterone, follicle‐stimulating hormone, and total antioxidant capacity decreased in female rats. Curcumin treatment restored serological indices toward normal levels (Elsawi et al., 2023). Despite of studies available on curcumin functions, a knowledge gap persists in the literature concerning the optimal curcumin dosage requisite for conferring protective effects against ACR‐induced ovarian damage. Studying dose–response relationships and assessing the long‐term effects of curcumin supplementation can help establish guidelines for effective and safe supplementation. Therefore, this study aimed at conducting a comprehensive investigation into the gene and protein expressions of first‐line antioxidant levels and apoptosis‐related factors during prolonged ACR exposure in female Balb/c mice. The objective was to provide insights into the effectiveness of curcumin, administered at two different doses, in alleviating ACR‐induced toxicity as a potential therapeutic strategy for maintaining ovarian health.

## 2 MATERIALS AND METHODS

### 2.1 Animal and treatment

A total of forty healthy female Balb/c mice weighing 20–25 g (6–8 weeks old) were purchased from the animal house at Shiraz University of Medical Sciences. All components of the approach that involved animals were authorized by the ethical committee at Shiraz University of Medical Sciences (IR.SUMS.REC.1400.199) and conform to the Institutional and National Guide for the Care and Use of Laboratory Animals.

Animals were housed in standard cages in a temperature‐controlled environment at 22–24°C using artificial light cycles (12 h light/12 h dark) and unlimited access to chow and water. After 14 days of adaptation, the animals were randomly allocated to four groups (*n* = 10/group) as follows: The control group received normal saline; ACR group received acrylamide (50 mg/kg); ACR/CUR100 received Acr (50 mg/kg) + curcumin (100 mg/kg); ACR/CUR200 received Acr (50 mg/kg) + curcumin (200 mg/kg). All reagents were given by oral gavage, once a day, for 35 days where the reagents were administered directly into the stomach using a precise dosing technique. This approach was chosen for its ability to ensure accurate dosage control and maintain a consistent exposure regimen. At the end of the experiments, animals were sacrificed by cervical dislocation. The right ovaries were kept at −80°C for gene expression analysis, and the left ovaries were preserved in buffer formalin for immunofluorescence assessments.

### 2.2 Gene expression analysis by real‐time polymerase chain reaction

Total RNA was extracted from three ovaries per group using RNX‐ Plus Solution (SinaClon, Iran; Cat. No. EX6101) according to the manufacturer's instructions. The RevertAid First Strand cDNA synthesis kit (Fermentas, Thermo Fisher Scientific, Waltham, MA, USA; Cat. No. K1622) was used to synthesize first‐strand cDNA according to the manufacturer's instructions. An ABI PRISM® 7500 Sequence Detection System was used to perform real‐time RT‐PCR (Applied Biosystems, Foster City, USA). 1 μL of cDNA template, 1 μL of each primer (10 pmol/L), and 12.5 μL of RealQ Plus 2× Master Mix Green low ROX (Amplicon, Odense, Denmark; Cat. No.: A324406) were used for PCR amplification in a final volume of 25 μL. The target gene dosage level was normalized using Beta‐Actin (*B.act*) as a reference gene. *Gpx*, *Sod*, *Cat*, *Bax*, *Bcl2l1*, and *Caspase 3* gene expression were investigated. The 2^−ΔΔCt^ technique was used to evaluate the samples. The primers used in RT‐PCR are mentioned in Table 1.

**TABLE 1:** Primer sequences used for real‐time PCR analysis.

Gene	Sequence forward (5′–3′)	Sequence reverse (5′–3′)	Product size (bp)
*Sod*	CGGATGAAGAGAGGCA	TGTACGGCCAATGATGGA	125
*Cat*	AGCTGATCACAGTTCGTGA	ATGGCATCCTGATGAAGA	111
*Gpx*	CCACCGTGTATGCCTTCTC	GGGACGCGACATTCTCAAT	102
*Bcl2l1*	GCAGGTATTGGTGAGTCGGA	CTCGGCTGCTGCATTGTTC	130
*Bax*	TGGAGATGAACTGGACAGCAAT	TAGCAAAGTAGAAGAGGGCAACC	155
*Caspase‐3*	TGACTGGAAAGCCGAAACTC	AGCCTCCACCGGTATCTTCT	122
*B.act*	TCCTGACCCTGAAGTACCC	CACACGCAGCTCATTGTAGA	98

### 2.3 Immunofluorescence staining of GPX, SOD, and CAT proteins in mice ovary

The left ovaries were fixed, dehydrated, and embedded in paraffin wax and serial sections (5 μm thickness) were prepared. The slice's deparaffinization was performed at 60°C for 30 min and hydrated. The sections were then microwaved in antigen unmasking solution, followed by rinsing in PBS, and incubated with Triton 100× (Sigma, UK) for 30 min at room temperature. The slices were rinsed in PBS and treated in 10% goat serum (Biowest, S2000‐100) for 30 min at room temperature to block nonspecific sites. The sections were then treated with primary antibodies against SOD in a wet chamber at 4°C overnight: orb67357 (orb688926), GPX: SC133160 (ORB688926), and CAT: ORB538719 (ORB688925) (1:100 dilution). The signals were observed the next morning in the dark with a secondary antibody (ab6785) (goat anti‐mouse IgG, 1:200 dilution). The nucleus of the cell was stained with DAPI (Sigma, UK, 5 g/mL), rinsed with PBS, and examined under a fluorescence microscope (Olympus, BX51, Japan).

The image analysis was carried out with the Nis‐Elements BR 3.0 system (Nikon). The total number of pixels for the total area assessed using color segmentation analysis was used to calculate immunofluorescence. The intensity of the fluorescence was measured using Image J software. The fluorescence emission for each sample was determined and then normalized based on the emission of the control group. Before assessing the statistical difference between the groups, background fluorescence values were eliminated from the final values. Image J software was used to calculate the relative fluorescence intensity in each sample.

### 2.4 TUNEL assay

To perform the TUNEL assay, the sections were initially subjected to de‐waxing and rehydration using decreasing ethanol concentrations, followed by a rinse with phosphate‐buffered saline (PBS). Subsequently, the specimens were immersed in a 50 μL proteinase K solution (comprising 1 μL proteinase K and 50 μL apoptosis‐grade water) for 10 minutes. Finally, the sections were washed in PBS and exposed to a 50 μL labeling reaction mixture (consisting of TUNEL enzyme solution and labeling solution in a 1:10 ratio) for 2 h at 37°C in a humid chamber and darkness (Khodabandeh et al., 2021).

### 2.5 Statistical analysis

Statistical tests were performed using the statistical package for the social sciences (SPSS version 24.0. SPSS Inc., Chicago, IL, USA). Data are reported as the means ± standard deviation (SD). Multiple group comparisons were performed using the one‐way analysis of variance (ANOVA) test followed by a post hoc Tukey test. The level of significance was set at *p* ≤ .05.

## 3 RESULTS

Different analyses were conducted to compare groups, including:

1. between the Acr‐received group and control;
2. between curcumin‐treated and control groups;
3. between curcumin‐treated and Acr‐received group.

### 3.1 Level of *Sod*, *Cat*, and *Gpx* genes expression

**FIGURE 1:**
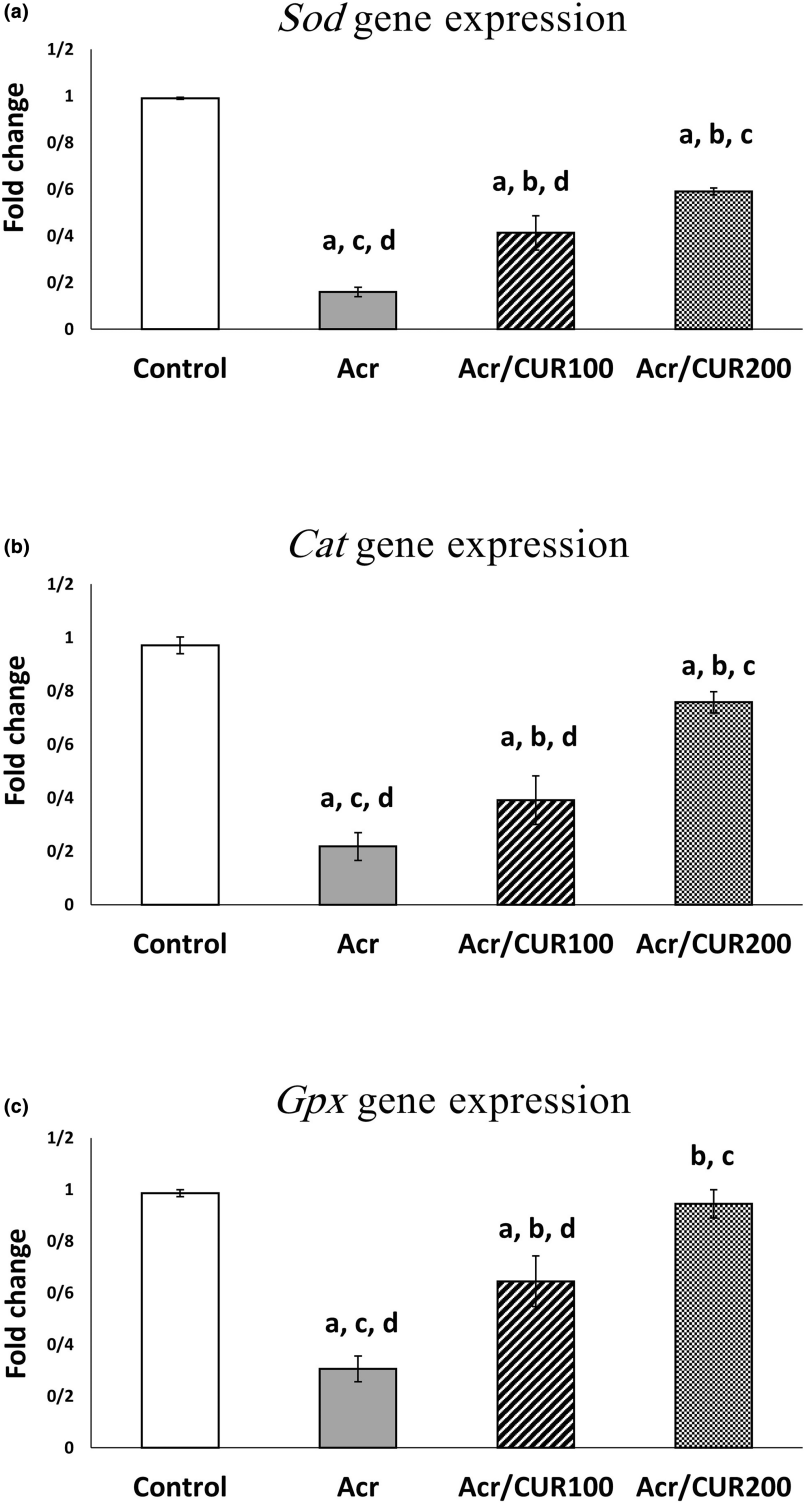
The relative expression of *Sod*, *Cat*, *and Gpx* genes at the mRNA level of ovaries in the curcumin treatment versus the untreated groups versus control. Bar represented by mean ± SD and the *p <* .05 was considered as significant. ^a^: vs. Control group. ^b^: vs. Acr group. ^c^: vs. Acr/CUR100 group. ^d^: vs. Acr/CUR200 group.

As shown in Figure 1 ^(a–c)^, the model group (Acr) showed decreased mRNA expression of *Sod*, *Cat*, and *Gpx* genes compared with the control group. Treatment with two different doses of curcumin (CUR100 and CUR200) significantly increased the expression of three studied genes compared to the Acr‐received group. Interestingly, the expression of the *Gpx* gene reached near to the control in the Acr/CUR200 group compared with the model and Acr/CUR100 groups (Figure 1 ^(c)^). In addition, the higher dose of curcumin (CUR200) showed significant recovery of the antioxidant gene expression compared to the CUR100 dose (Figure 1 ^(a–c)^).

### 3.2 Level of *Sod*, *Cat*, and *Gpx* protein expression

**FIGURE 2:**
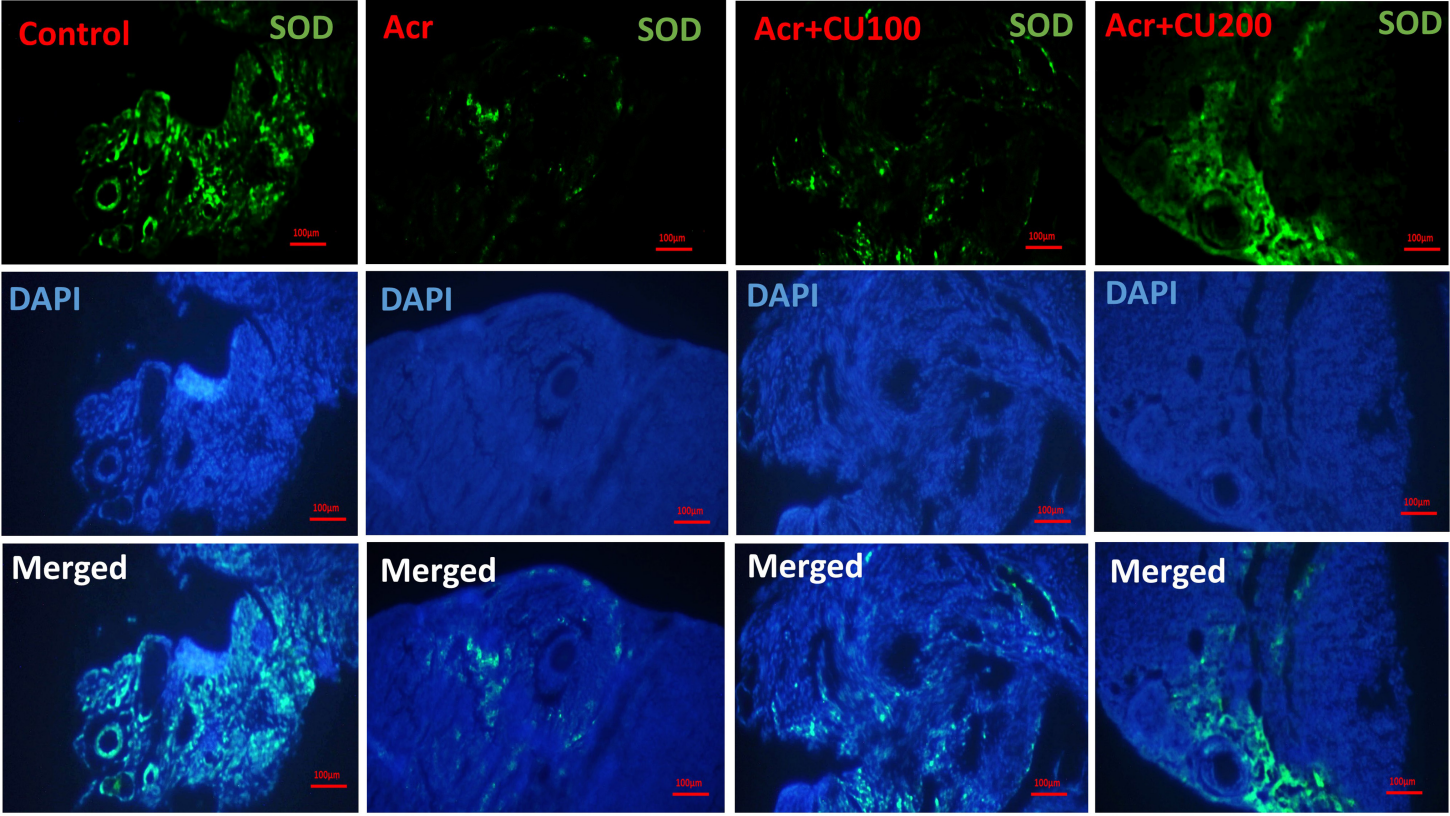
Representative pictures of immunofluorescence staining for SOD (shiny green) proteins in ovarian sections of mice, counterstained with DAPI for nuclei (blue), and merged pictures, in control, acrylamide (Acr), Acrylamid+curcumin100 (Acr/CUR100), and Acrylamid+curcumin200 (Acr/CUR200) groups.

**FIGURE 3:**
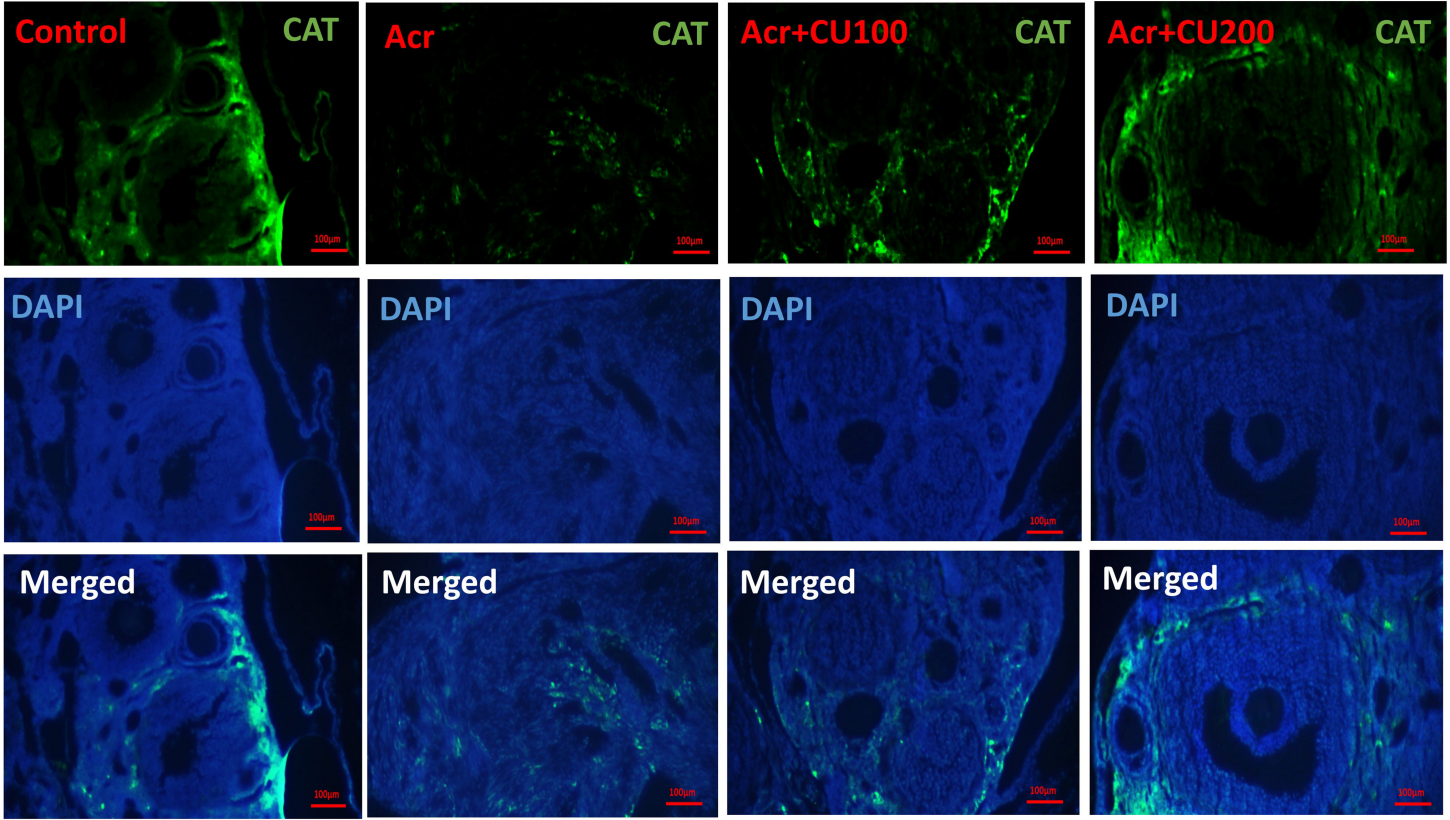
Representative pictures of immunofluorescence staining for CAT (shiny green) proteins in ovarian sections of mice, counterstained with DAPI for nuclei (blue), and merged pictures, in control, acrylamide (Acr), Acrylamid+curcumin100 (Acr/CUR100), and Acrylamid+curcumin200 (Acr/CUR200) groups.

**FIGURE 4:**
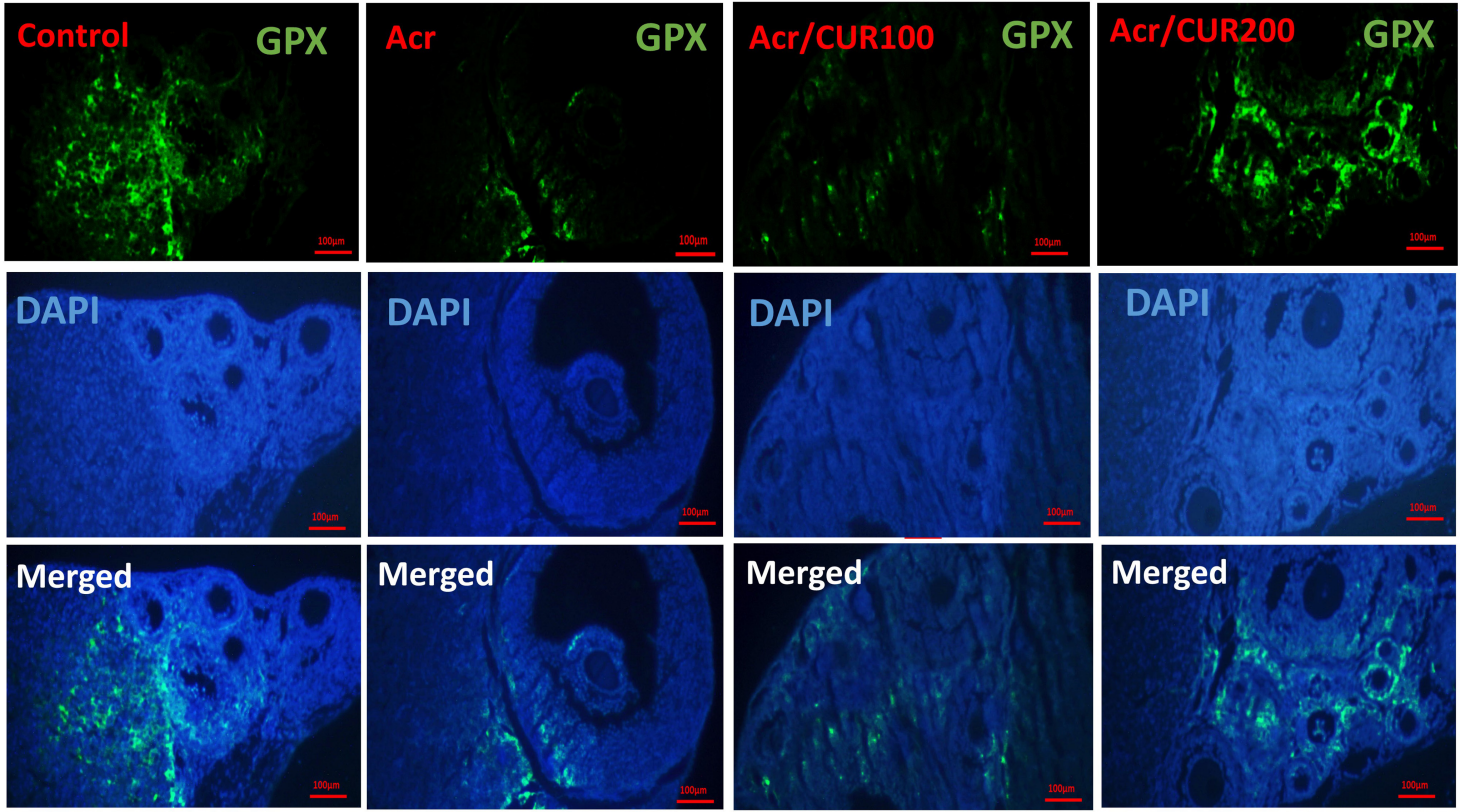
Representative pictures of immunofluorescence staining for GPX (shiny green) proteins in ovarian sections of mice, counterstained with DAPI for nuclei (blue), and merged pictures, in control, acrylamide (Acr), Acrylamid+curcumin100 (Acr/CUR100), and Acrylamid+curcumin200 (Acr/CUR200) groups.

The representative pictures of immunofluorescence staining for SOD, CAT, and GPX protein expression in the ovary of the studied groups were shown in Figures 2, 3, 4, respectively.

**FIGURE 5:**
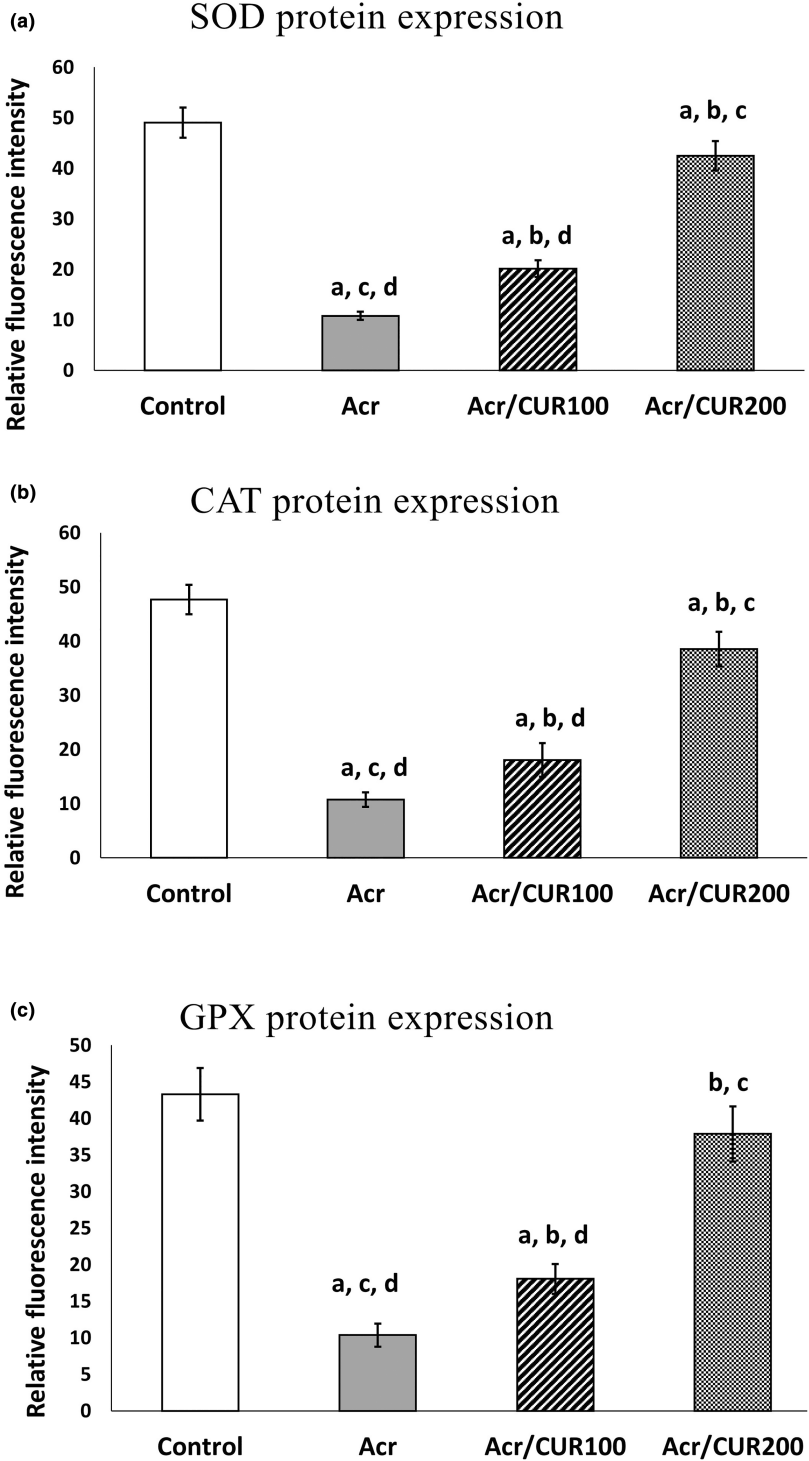
Quantification of Immunofluorescence staining for SOD, CAT, and GPX protein expression. Data expressed as means ± SD. Data are statistically significant at *p <* .05. ^a^: vs. Control group. ^b^: vs. Acr group. ^c^: vs. Acr/CUR100 group. ^d^: vs. Acr/CUR200 group.

In the ovary of mice from the Acr‐received group, SOD, CAT, and GPX were significantly reduced compared to the control group. Furthermore, as shown in Figure 5 ^(a–c)^, the protein expression of three investigated genes revealed the same trend as mRNA gene expression after treatment with two doses of curcumin (CUR100 and CUR200), so that significantly increased compared to the Acr‐received group. The protein expression of the GPX gene reached near to the control in the Acr/CUR200 group compared with the other two groups (Figure 5 ^(c)^).

### 3.3 Level of Bax, Bcl2l1, and *Caspase‐3* genes expression

Compared to the control group, the gene expression of *Bax* showed a significant increase in the Acrylamide group. However, in both acrylamide groups that received Curcumin, the expression was significantly reduced compared to the acrylamide‐only group. The expression was comparable between both acrylamide+curcumin groups and the control group.

In contrast, the gene expression of *Bcl2l1* significantly decreased in the Acrylamide group compared to the control group. Although its expression increased in both Curcumin‐received groups compared to the acrylamide group, the difference was not statistically significant. Notably, the expression in the Acr‐CUR100 group was significantly lower than in the control group.

**FIGURE 6:**
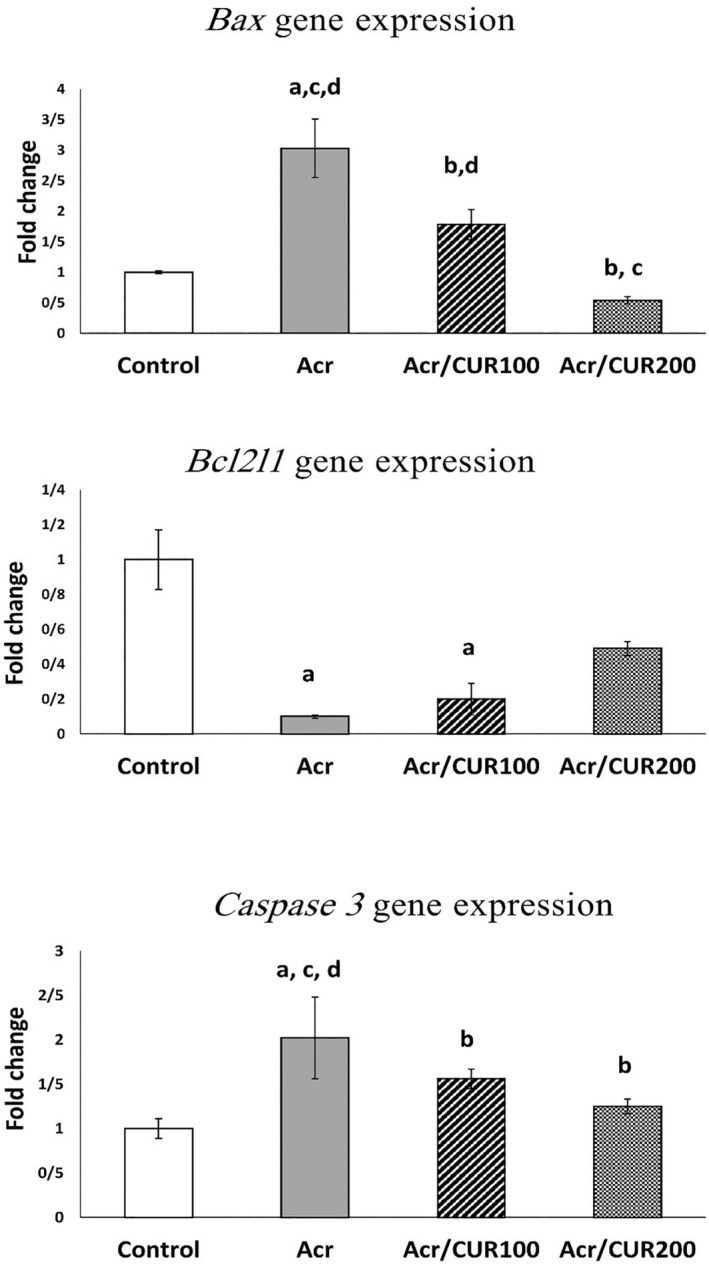
The relative expression of *Bax*, *Bcl2l1*, *and Caspase 3* genes at the mRNA level of ovaries in the curcumin treatment versus the untreated groups versus control. Bar represented by mean ± SD and the *p <* .05 was considered as significant. ^a^: vs. Control group. ^b^: vs. Acr group. ^c^: vs. Acr/CUR100 group. ^d^: vs. Acr/CUR200 group.

Similarly, the gene expression of *Caspase 3* significantly increased in the Acrylamide group, but it significantly decreased in the Acr‐CUR100 and Acr‐CUR200 received groups compared to the acrylamide group. The expression remained similar in both curcumin groups and the control group when compared to each other (Figure 6).

### 3.4 Apoptosis in the ovaries

**FIGURE 7:**
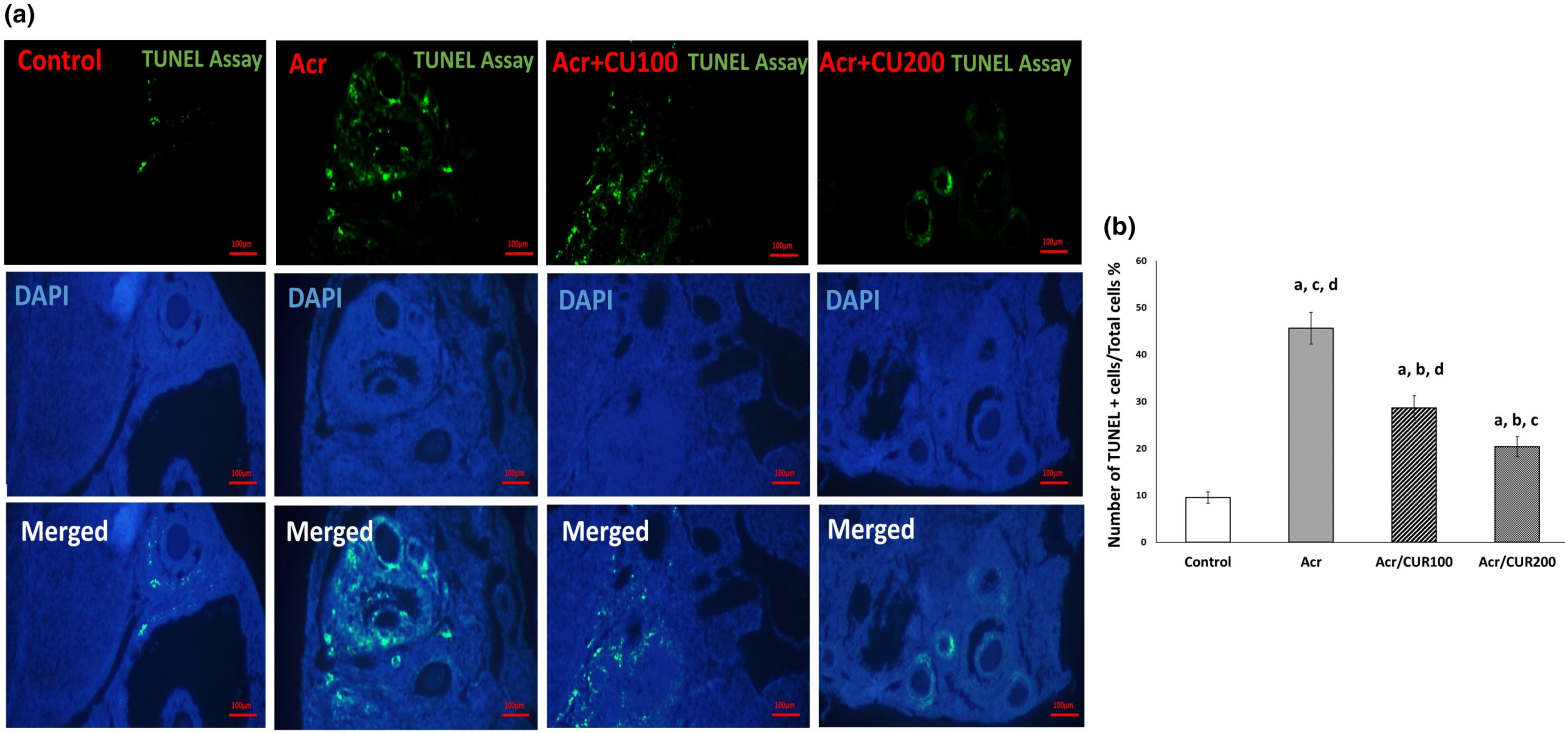
(a) Representative pictures of immunofluorescence staining for TUNEL (green fluorescence) in ovarian sections of mice, counterstained with DAPI for nuclei (blue), and merged pictures, in control, acrylamide (Acr), Acrylamid+curcumin100 (Acr/CUR100), and Acrylamid+curcumin200 (Acr/CUR200) groups. (b) Quantification of Immunofluorescence staining for SOD, CAT, and GPX protein expression. Data expressed as means ± SD. Data are statistically significant at *p <* .05. ^a^: vs. Control group. ^b^: vs. Acr group. ^c^: vs. Acr/CUR100 group. ^d^: vs. Acr/CUR200 group.

Findings of quantitative analysis of apoptotic cells in the ovaries showed that in the Acrylamide group, the level of apoptotic cells increased significantly as compared to the control group. However, in those who received curcumin 100 and 200 its level markedly decreased in comparison to the acrylamide group (Figure 7 ^(a‐b)^).

## 4 DISCUSSION

The aim of this study was to study how curcumin influences ACR‐induced changes in the mice ovarian tissue. Exposure to ACR led to a decrease in the mRNA expression of antioxidant genes. However, administering CUR100 and CUR200 significantly increased gene expression, as reflected in raised levels of SOD, CAT, and GPX proteins. Furthermore, ACR exposure raised the levels of pro‐apoptotic markers shown by increase in rate of apoptosis. In contrast, curcumin markedly decreased pro‐apoptotic markers and apoptotic cells, emphasizing its potential role in alleviating ACR‐induced apoptosis. Notably, CUR200 exhibited a more substantial recovery, highlighting its effectiveness in mitigating ACR‐induced reproductive toxicity.

Reproductive toxicity resulting from ACR exposure involves a crucial aspect—the alteration of antioxidant defense mechanisms. Antioxidant molecules play a pivotal role in neutralizing and scavenging radicals, as well as repairing damage caused by them. These antioxidants are categorized into four types based on their defense mechanisms: first‐line, second‐line, third‐line, and fourth‐line defense. Specifically, SOD, CAT, and GPX, the focal points of this investigation, belong to the first‐line defense antioxidant category (Bakhtari et al., 2020; Ighodaro & Akinloye, 2018). These enzymes, namely SOD, CAT, and GPX, work to convert harmful superoxide anion (*O2), hydrogen peroxides (H_2_O_2_), hydroperoxide, and lipid peroxides into less harmful and non‐toxic molecules, such as H_2_O, O_2_, and corresponding alcohols (Alaee et al., 2023; Ighodaro & Akinloye, 2018).

Reproductive toxicity induced by ACR has been extensively documented in various studies. Consistent with our findings, Hamdy et al. (2017) reported significant decreases in ovarian catalase (Cat) activity, superoxide dismutase (SOD) activity, reduced glutathione (GSH) levels, and glutathione peroxidase (GSH‐Px) activity in female rats exposed to ACR (Hamdy et al., 2017). Wang et al. demonstrated that acrylamide induces oxidative stress, resulting in reduced nitric oxide (NO) levels, with observed consequences including the inhibition of cell growth and the induction of cell death (Wang et al., 2023).

Furthermore, Seberi et al. illustrated a noteworthy increase in malondialdehyde (MDA) concentration and a significant decrease in total antioxidant capacity in the blood of female NMRI mice subjected to ACR for a duration similar to our study (Saberi et al., 2022). These findings emphasize the impact of ACR on antioxidant mechanisms and underscore the significance of exploring potential protective interventions.

Various antioxidant treatment modalities, including the utilization of ascorbic acid (Firouzabadi et al., 2022), curcumin, and taurine (Saberi et al., 2022), as well as thymoquinone (Huwait et al., 2023), have the potential to mitigate the apoptotic and toxic effects resulting from ACR intake on reproductive tissue.

Our findings indicate that the administration of curcumin at doses of 100 and 200 mg/kg efficiently restores the expression of antioxidant genes, demonstrating a significant improvement at the 200 mg/kg dose compared to the 100 mg/kg dose. Several studies have illustrated the efficacy of curcumin in mitigating oxidative stress‐related concerns, encompassing factors like total antioxidant capacity, malondialdehyde (MDA), and superoxide dismutase (SOD) within physiological conditions (Chen et al., 2022; Khayatan et al., 2023). In a recent systematic review and meta‐analysis of randomized controlled trials, curcumin exhibited a substantial influence on indicators of oxidative stress, including total antioxidant capacity, malondialdehyde, and SOD levels (Dehzad et al., 2023).

In various animal models of ovarian diseases, curcumin has demonstrated the ability to enhance overall ovarian function (Eser et al., 2015; Wang et al., 2017). Furthermore, the administration of curcumin has been proven beneficial in addressing gynecological diseases in women, as highlighted in studies (Kamal et al., 2021; Shen et al., 2022). For instance, curcumin supplementation has beneficial effects on weight loss, glucose and lipid metabolism, metabolic parameters, and androgen levels in polycystic ovary syndrome patients (Ghanbarzadeh‐Ghashti et al., 2023; Sohaei et al., 2019). Curcumin causes a reduction in serum nitric oxide release in women with primary dysmenorrhea and premenstrual syndrome pain (Farrokhfall et al., 2023). It also decreases the total score of primary symptoms of menopause by affecting oxidative Stress Biomarkers (Farshbaf‐Khalili et al., 2022).

Bax, Bcl2l1 (Bcl‐2), and Caspase 3 are crucial regulators in the apoptotic pathway, playing pivotal roles in determining cell fate. Specifically, Bax and Caspase 3 promote apoptosis, whereas Bcl2l1 counteracts these effects. The delicate balance among these factors serves as a key determinant in cellular survival and programmed cell death (Czabotar & Garcia‐Saez, 2023). Our study showed significant alterations in gene expression related to apoptosis within the ovarian tissues of mice exposed to ACR. The observed increase in Bax expression in the Acrylamide group suggests a pro‐apoptotic shift, highlighting the potential adverse impact of acrylamide on ovarian health.

The introduction of curcumin in both acrylamide‐treated groups resulted in a significant reduction in Bax expression, effectively attenuating the pro‐apoptotic effects induced by ACR. Conversely, the downregulation of Bcl2l1 in the ACR group suggests a suppression of anti‐apoptotic signals, further supporting the pro‐apoptotic environment. Although curcumin supplementation exhibited an increasing trend in Bcl2l1 expression compared to the acrylamide group, statistical significance was not achieved. Notably, the Acr‐CUR100 group demonstrated a significantly lower expression of Bcl2l1 than the control group, suggesting a potential dose‐dependent response to curcumin.

The dynamics of Caspase 3 expression revealed a noteworthy pattern. The ACR group exhibited a significant increase in Caspase 3 expression, indicative of enhanced apoptotic activity. However, the ACR‐CUR100 and ACR‐CUR200 groups displayed a significant decrease in Caspase 3 expression, emphasizing the protective role of curcumin against acrylamide‐induced apoptosis. Interestingly, the expression levels of Caspase 3 in both curcumin groups were comparable to those in the control group, suggesting that curcumin effectively counteracts the apoptotic effects induced by acrylamide.

The gene expression results of Bax, Caspase 3, and Bcl2l1 were corroborated by the TUNEL assay. In the Acrylamide group, a significant elevation in apoptotic cell levels was observed. However, the administration of curcumin at doses of 100 and 200 demonstrated a remarkable reduction in apoptotic cell levels. These outcomes align with Firouzabadi et al.'s findings, where they noted alterations in the proportions of apoptosis‐related genes (pro‐apoptotic to anti‐apoptotic) in mice orally exposed to acrylamide (10 mg/kg), resulting in ovarian dysfunction (Firouzabadi et al., 2022). Additionally, a separate study reported a notable increase in ovarian follicle apoptosis after oral acrylamide administration (Aldawood et al., 2020).

Consistent with these results, Elsawi et al. found that administering acrylamide (20 mg/kg b.w.) to female rats for 21 days reduced serum Total Antioxidant Capacity, leading to ovarian follicle regression. However, curcumin (100 mg/kg b.w.) administration reversed these effects (Elsawi et al., 2023). The intraperitoneal administration of curcumin at a dose of 100 mg/kg to mice with d‐gal‐induced premature ovarian failure resulted in a reduction of serum oxidative stress and apoptosis in the ovarian tissue, effectively preserving ovarian function (Yan et al., 2018).

In a different study, acrylamide induced decidua apoptosis in the endometrium by up‐regulating Bax and cleaved‐caspase‐3, while simultaneously decreasing Bcl‐2 protein levels (Yu et al., 2020). In light of these findings, curcumin emerges as a potential remedy for ovary‐related disorders owing to its anti‐apoptotic, anti‐inflammatory, and antioxidant properties (Kamal et al., 2021).

**FIGURE 8:**
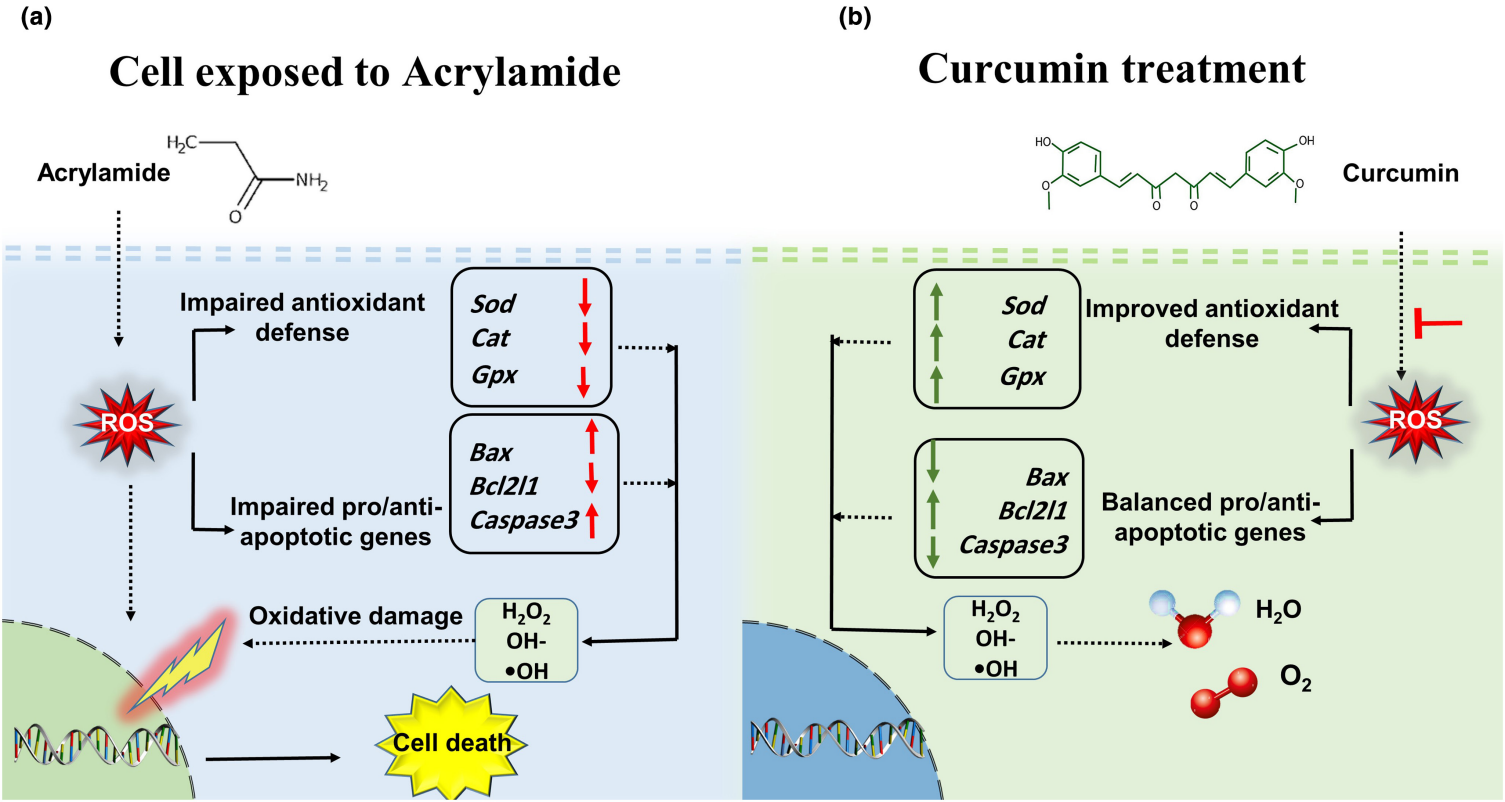
(a) Schematic representation of the impact of acrylamide exposure on ovarian tissue. (b) The therapeutic potential of curcumin in restoring antioxidant balance and mitigating toxicity in female mice.

Figure 8 illustrates the intricate dynamics of acrylamide exposure on ovarian tissue, showcasing the consequential effects on ovarian antioxidant defense. Subsequently, an evident imbalance in pro‐ and anti‐apoptotic markers is portrayed, highlighting the potential mechanisms contributing to acrylamide‐induced toxicity in the ovaries of female mice (Figure 8a). In contrast, Panel b of Figure 8 visually represents the promising therapeutic intervention provided by curcumin treatment. This intervention effectively restores the ovarian antioxidant potential, as evidenced by the harmonized expression of pro‐ and anti‐apoptotic markers, demonstrating curcumin's potential in mitigating acrylamide‐induced toxicity in the ovarian microenvironment.

## 5 CONCLUSION

Our findings highlight the adverse impact of ACR on ovarian antioxidant defense, leading to an imbalance in pro‐ and anti‐apoptotic markers. Importantly, curcumin treatment emerged as a promising therapeutic intervention, effectively restoring antioxidant potential and mitigating acrylamide‐induced toxicity in the ovaries of female mice. These results contribute to a deeper understanding of the molecular mechanisms involved in ACR‐induced reproductive toxicity and underscore the potential of curcumin as a protective agent against such detrimental effects.

## AUTHOR CONTRIBUTIONS

**Sanaz Alaee:** Conceptualization (equal); data curation (equal); formal analysis (equal); funding acquisition (equal); investigation (equal); methodology (equal); writing – original draft (lead); writing – review and editing (equal). **Zahra Khodabandeh:** Conceptualization (equal); data curation (equal); funding acquisition (equal); methodology (equal); supervision (equal); writing – review and editing (equal). **Mahintaj Dara:** Data curation (equal); formal analysis (equal); investigation (equal); writing – review and editing (equal). **Elham Hosseini:** Conceptualization (equal); data curation (equal); formal analysis (equal); validation (equal); writing – original draft (equal); writing – review and editing (equal). **Mona Sharma:** Formal analysis (equal).

## FUNDING INFORMATION

The study has been supported by the Shiraz University of Medical Sciences, Shiraz, Iran; (Project number: 23162).

## CONFLICT OF INTEREST STATEMENT

The authors declare that they have no competing interests.

## ETHICS STATEMENT

The experimental protocol of the animal study was approved by the ethical committee of Shiraz University of Medical Sciences, Shiraz, Iran (IR.SUMS.REC.1400.199).

## Data Availability

All data generated or analyzed during this study are included in the manuscript.
